# Sex Disparity in the Association of Metabolic Syndrome with Cognitive Impairment

**DOI:** 10.3390/jcm13092571

**Published:** 2024-04-27

**Authors:** Yi-Min Chang, Chia-Lin Lee, Jun-Sing Wang

**Affiliations:** 1 Department of Internal Medicine, Taichung Veterans General Hospital, Taichung 407219, Taiwan; ymchang1995@gmail.com; 2 Intelligent Data Mining Laboratory, Department of Medical Research, Taichung Veterans General Hospital, Taichung 407219, Taiwan; u502107@yahoo.com.tw; 3 Department of Medicine, School of Medicine, National Yang-Ming Chiao Tung University, Taipei 112304, Taiwan; 4 Division of Endocrinology and Metabolism, Department of Internal Medicine, Taichung Veterans General Hospital, No. 1650, Sec. 4, Taiwan Boulevard, Taichung 407219, Taiwan; 5 Department of Post-Baccalaureate Medicine, College of Medicine, National Chung Hsing University, Taichung 402202, Taiwan

**Keywords:** cognitive impairment, dementia, metabolic syndrome, mini-mental state examination, sex disparity

## Abstract

**Background/Objectives:** Metabolic syndrome (MS) is a constellation of several cardiometabolic risk factors. We investigated sex disparity in the associations between MS and cognitive impairment using cross-sectional data from Taiwan Biobank. **Methods:** We determined the associations of MS and its five components with cognitive impairment (mini-mental state examination, MMSE < 24) and the five domains of MMSE using logistic regression analyses. **Results:** A total of 7399 men and 11,546 women were included, and MS was significantly associated with cognitive impairment only in women (adjusted OR 1.48, 95% CI 1.29–1.71, *p* = 0.001) (p for interaction 0.005). In women, the association with MS was significant in orientation (adjusted OR 1.21, 95% CI 1.07–1.37, *p* = 0.003), memory (adjusted OR 1.12, 95% CI 1.01–1.25, *p* = 0.034) and design copying (adjusted OR 1.41, 95% CI 1.23–1.62, *p* = 0.001) (*p* value for interaction 0.039, 0.023, and 0.093, respectively). Among the components of MS, a large waist circumference (adjusted OR 1.25, 95% CI 1.08–1.46, *p* = 0.003), high fasting glucose (adjusted OR 1.16, 95% CI 1.00–1.34, *p* = 0.046), and low HDL cholesterol (adjusted OR 1.16, 95% CI 1.00–1.34, *p* = 0.049) were significantly associated with cognitive impairment in women. **Conclusions:** Our findings suggest that sex has a significant influence on the association between MS and cognitive dysfunction, especially in orientation and memory.

## 1. Introduction

In 2020, the prevalence estimates of dementia, regardless of the type, was 58.66 million worldwide according to the World Alzheimer Report 2015 [1], with Asia accounting for the highest prevalence (29.23 million, 49.8%). As a consequence of population growth and population aging, the prevalence of dementia has been projected to rise to 82 million cases by 2030 and 152.8 million cases by 2050 [2,3]. The escalated trends in the number of dementia patients, especially in low- and middle-income countries, have imposed a heavy burden on healthcare expenditure and socio-economic development.

In this regard, the 2020 Lancet Commission on dementia prevention, intervention, and care proposed 12 potentially modifiable risk factors [4], including hypertension, obesity, diabetes, hearing impairment, smoking, excessive alcohol consumption, traumatic brain injury, depression, physical inactivity, low education level, social isolation contact, and air pollution. This study emphasized that not only treating medical diseases to improve physical health but also keeping cognitively and socially active are of great importance in the prevention of developing dementia. Besides the above risk factors, dyslipidemia had been pointed out to have a close relationship with cognitive decline [5,6,7,8,9], indicating that higher triglyceride and lower high-density lipoprotein (HDL) cholesterol predicted poor cognition performance. Metabolic syndrome, as a constellation of more than three metabolic risk factors, is associated with a higher susceptibility of developing cognitive impairment and deteriorating, leading to dementia [8,9,10,11,12,13]. A meta-analysis in 2018 [14] reported that people with metabolic syndrome were about five times more likely to have cognitive deterioration from mild cognitive impairment to dementia. However, the influence of metabolic syndrome on different domains of cognition remains unclear [15,16]. In a cross-sectional study [16], MS was associated with poor memory performance, but not speed of reaction.

Several studies [17,18,19,20] have demonstrated a sex disparity in the prevalence of metabolic syndrome. Metabolic syndrome was more prevalent in men than in pre-menopausal women [18]; however, the prevalence reversed when comparing men with post-menopausal women [19,20]. Moreover, women seemed to be more susceptible to develop dementia than men, and the susceptibility become more prominent with increasing age [21,22,23]. The EURODEM study [23] including people 65 years and older reported that women had a higher cumulative risk of developing dementia than men, and that there was a significant increase in the incidence of dementia after 85 years of age in women compared with men. Given that both metabolic syndrome and dementia had a higher prevalence in elderly women, there might be gender differences in the association between metabolic syndrome and dementia.

In addition, a low education level had been recognized as an independent risk factor for both metabolic syndrome and cognitive impairment [24,25]. The Women’s Health in the Lund Area (WHILA) study found that women with higher educational attainment had a lower prevalence of metabolic syndrome [24]. Another study including 6314 women aged 66 years or older showed that the higher education attainment, the better cognitive performance and lower risk of cognitive decline [25]. Lower education attainment has been reported to be associated with a higher prevalence of metabolic syndrome and higher risk of cognitive decline independently. The effect of education level on the association between metabolic syndrome and cognitive impairment needs further evaluation.

In this cross-sectional study using data from Taiwan Biobank, we aimed to assess the sex disparity and the impact of education level on the association between metabolic syndrome and cognitive impairment and to investigate the gender-specific effect on the association of each component of metabolic disorders with different domains of the mini-mental state examination (MMSE) [26].

## 2. Materials and Methods

### 2.1. Study Participants

Taiwan Biobank [27] is a Taiwanese-based database, aimed at collecting information about whole genome sequences, identifying specific genetic mutations, recognizing risk factors of a variety of common diseases, exploring novel and advanced therapeutic strategies, and improving the health of the general population. The information was collected by health examination, laboratory testing, and questionnaires from community-dwelling subjects and patients in hospitals. All participants provided informed consent. This study was approved by the Institutional Review Board of Taichung Veterans General Hospital, Taichung, Taiwan (approval number CE20023A), and was conducted in accordance with the Declaration of Helsinki.

### 2.2. Cognitive Assessment and the Definition of Dementia

MMSE, the most common instrument for screening dementia in clinical practice, was used to evaluate the cognitive function of study participants. Its Chinese version has been validated and used for research [28,29]. People were investigated face to face by well-trained investigators, and the administration of MMSE took about 5 to 10 min. MMSE [26] composed of 11 questions/tasks with a maximum score of 30 points, including the following five domains: (a) orientation (maximum 10 points), (b) memory (maximum 6 points), (c) attention and calculation (maximum 5 points), (d) language (maximum 8 points), and (e) design copying (constructional praxis) (maximum 1 point). The higher the MMSE scores, the better the cognitive function. In this study, an MMSE score < 24 was defined as cognitive impairment [30]. For the five domains of MMSE, participants who scored zero points in design copying or scored two points less than the maximum score of the other domain were considered to have impairment in that cognitive function [31].

### 2.3. Definition of Metabolic Syndrome

Metabolic syndrome was determined according to the criteria of the National Cholesterol Education Program (NCEP) Adult Treatment Panel III (ATP III) guidelines [32]. Participants were considered as having metabolic syndrome if they had at least three of the following components [33,34]: (a) a waist circumference greater than 90 cm in men or 80 cm in women, (b) hypertriglyceridemia (triglyceride ≥ 150 mg/dL), (c) high-density lipoprotein (HDL) cholesterol less than 40 mg/dL in men or less than 50 mg/dL in women, (d) high blood pressure (systolic pressure ≥ 130 mm Hg or diastolic pressure ≥ 85 mm Hg), and (e) high fasting plasma glucose (≥100 mg/dL) or previously diagnosed type 2 diabetes.

### 2.4. Statistical Analysis

**Figure 1:**
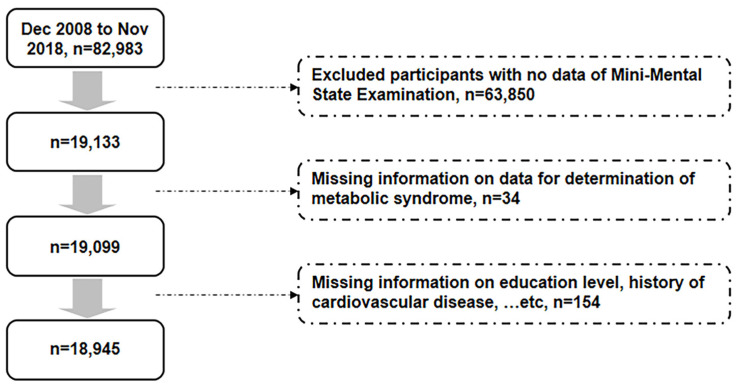
Flow diagram displaying the selection of study participants for analyses.

Participants were enrolled from Taiwan biobank from December 2008 to November 2018, and a total of 82,983 participants were enrolled (Figure 1). For complete data collection and precise data analysis, participants with missing data on MMSE (*n* = 63,850), parameters for the diagnosis of metabolic syndrome (*n* = 34), or other relevant information (*n* = 154) were excluded from the analyses. Finally, a total of 18,945 participants were analyzed in this study. Data are presented as mean (95% CI) and numbers (percentages), respectively, for continuous and categorical variables. First, logistic regression analyses were applied to determine the association of metabolic syndrome with cognitive impairment in the overall population, different genders and people with different education levels. Then, the interaction in logistic regression was examined to investigate the influence of sex and different education level on the association between metabolic syndrome and cognitive impairment after being adjusted for age, smoking and cardiovascular diseases. Furthermore, we investigated the associations of metabolic syndrome and its five components with cognitive impairment (MMSE < 24) and the five domains of MMSE using logistic regression analyses, and the aforementioned associations were examined in subgroups of sex (male and female) independently. The goodness of fit of the logistic regression models was tested using the Hosmer–Lemeshow test in the subgroups. All the statistical analyses were conducted using the Statistical Package for the Social Sciences (IBM SPSS version 22.0; International Business Machines Corp., New York, NY, USA). A two-sided *p* value < 0.05 was considered statistically significant.

## 3. Results

Of the 18,945 subjects analyzed, 7399 were male with a mean age of 64.2 years, and 11,546 were female with a mean age of 63.7 years. Males had a worse metabolic condition (wider waist circumference, higher blood pressure, higher fasting plasma glucose and worse lipid profile), higher prevalence of chronic diseases (hypertension, diabetes mellitus, and cardiovascular disease), higher education level and better performance on MMSE than females (Table 1). The percentage of participants with an MMSE < 24 was lower in males than in females (5.4% vs. 9.0%, *p* < 0.001). Although the prevalence of metabolic syndrome was similar among males and females (30.3% vs. 30.4%, *p* = 0.905), there were significant between-group differences in the individual components of metabolic syndrome.

In the overall population, metabolic syndrome was significantly associated with cognitive impairment (adjusted OR 1.27, 95% CI 1.13 to 1.43, *p* < 0.001, Table 2). The association of metabolic syndrome with cognitive impairment was significant in females (adjusted OR 1.48, 95% CI 1.29 to 1.71, *p* < 0.001), but not in males (adjusted OR 1.02, 95% CI 0.82 to 1.27, *p* = 0.867; p for interaction 0.005). The *p* values of the Hosmer–Lemeshow test for the two subgroups were 0.282 and 0.886, respectively. The association between metabolic syndrome and cognitive impairment was more prominent in study participants with an education level ≤6 years (adjusted OR 1.21, 95% CI 1.03 to 1.41, *p* = 0.020), compared with those with an education level >6 years (adjusted OR 1.06, 95% CI 0.87 to 1.27, *p* = 0.579) (p for interaction 0.331). The respective *p* values of the Hosmer–Lemeshow test were 0.090 and 0.518.

Among the five components of metabolic syndrome, none had a significant association with cognitive impairment in the male participants (Table 3). In contrast, a large waist circumference (adjusted OR 1.25, 95% CI 1.08 to 1.46, *p* = 0.003), high fasting glucose (adjusted OR 1.16, 95% CI 1.00 to 1.34, *p* = 0.046), and low HDL cholesterol (adjusted OR 1.16, 95% CI 1.00 to 1.34, *p* = 0.049) were significantly associated with cognitive impairment in females. There was no gender-specific statistical significance in the association between each component of metabolic syndrome with cognitive impairment. The associations of metabolic syndrome and its components with five domains of MMSE were examined and are shown in Table 4. Metabolic syndrome was associated with impaired orientation (adjusted OR 1.21, 95% CI 1.07 to 1.37, *p* = 0.003), impaired memory (adjusted OR 1.12, 95% CI 1.01 to 1.25, *p* = 0.034), and impaired design copying (adjusted OR 1.41, 95% CI 1.23 to 1.62, *p* < 0.001) in women, but not in men (*p* value for interaction 0.039, 0.023, and 0.093, respectively). In women, impaired orientation and impaired design copying were significantly associated with each component of metabolic syndrome. In women, low HDL cholesterol was significantly associated with each domain of MMSE except for attention and calculation only; high fasting glucose was significantly associated with all domains of MMSE except for memory. Among the five components of metabolic syndrome, a large waist circumference, high fasting glucose, and low HDL cholesterol were associated with impairments of three domains of MMSE in women. When compared with men, there was a significant association in women between high triglyceride levels and impaired orientation (adjusted OR 1.23, 95% CI 1.08 to 1.42, *p* = 0.003, *p* value for interaction 0.025) and between high blood pressure and impaired orientation and calculation in women (adjusted OR 1.09, 95% CI 0.99 to 1.20, *p* = 0.068, *p* value for interaction 0.038).

## 4. Discussion

In this cross-sectional study involving 18,945 participants, we demonstrated sex disparities in the association between metabolic syndrome and cognitive impairment. Metabolic syndrome was significantly associated with cognitive impairment (MMSE < 24) in women only, especially in the orientation domain and memory domain of MMSE. Among the five components of metabolic syndrome, a large waist circumference, high fasting glucose, and low HDL cholesterol were significantly associated with impairments of more than three domains of MMSE in women, but not in men. Most of the previous studies [4,7,8,10,11,12,13,35,36,37] focus on the impact of metabolic disorders on cognitive decline and advocate lifestyle modifications or modifying metabolic and cardiovascular diseases to maintain cognitive function. Our findings suggest that sex difference plays an important role in the association of metabolic syndrome with cognitive impairment and deserves more attention in precise medical management of this global health issue.

Metabolic syndrome has been proven to have detrimental effects on cognitive function [7,8,9,10,11,12,13,14]. Hyperglycemia with insulin resistance is thought to be of great importance in the pathogenesis of neurodegenerative diseases. Prolonged peripheral hyperinsulinemia influences the expression of insulin receptors in brain microvasculature, attenuates insulin signaling in the central nervous system [38] and stimulates amyloid-β precursor protein accumulation [39], resulting in a decrease in regional glucose metabolism and predisposing patients to cognitive deficit. Moreover, cholesterol, as an essential component of cell membranes, modulates enzymes in the biosynthesis of amyloid-β protein, and dyslipidemia results in the accumulation of amyloid-β protein in the brain [40], which was associated with neurodegeneration and cognitive impairment. The deleterious effects of dyslipidemia on cognitive function have also been reported for atherosclerosis in cerebrovascular disease [41], which was also inseparable from hypertension. Hypertension causes changes in cerebrovascular vasculature not only due to atherosclerosis but also via the hypertrophic remodeling of smooth muscle cells [42], and these changes result in a reduction in the vascular diameter and increase the risk of cerebrovascular diseases. In addition, neurovascular coupling in response to increased neural activity and the endothelial function in regulating microvascular flow and anti-thrombosis formation are also disrupted due to hypertension. The decrease in cerebral blood flow with a mismatch between the demand of neural activity and blood supply contributes to poor cognitive performance. LK McEvoy et al. suggested that people with more components of metabolic syndrome indicated more defective cognitive function [43]. Jennifer L. Dearborna also reported that the more components of metabolic syndrome, the lower the performance in execution and word fluency [44]. Our study results were in agreement with the current understanding [8,9,10,11,12,13,14,45,46,47] that metabolic syndrome is significantly associated with cognitive impairment. Furthermore, we recognized that a large waist circumference, high fasting glucose and low HDL cholesterol had a significant influence on cognitive impairment, and this association was observed in women only.

Education is of great importance in improving people’s cognitive function, ameliorating their socioeconomic position, narrowing the disparity in health care access [48] and, therefore, relieving the disease burden of both cognitive decline and metabolic syndrome on global health. A study composed of 18,056 adult participants in the United States [49] found that education improved people’s performance on MMSE and narrowed the distribution of MMSE scores in the population. People with an education level less than 4 years had a median MMSE score of 22, but the median MMSE score increased to 29 in people with an education level of least 9 years. To preserve cognitive function and prevent developing dementia, Peeters Geeske emphasized the importance of education in older adults [50]. In addition, with education, it would be possible to understand the impact of metabolic diseases on people’s health, which in turn facilitates lifestyle modification, improves the cost-effectiveness of medical treatment and reduces the prevalence of metabolic syndromes. Anna Marseglia et al. [51] reported the protective effect of education on metabolic-syndrome-related cognitive decline. People with metabolic syndrome had poorer performance in cognitive function, especially in those with higher educational attainment. In people with metabolic syndrome, a higher education level was associated with better cognitive performance. Our study found that the association between metabolic syndrome and cognitive impairment was more prominent in participants with lower educational achievement, which was in accordance with previous studies.

Mild cognitive impairment is referred to as having subjective or objective memory impairment but without defects in activities of daily life or being diagnosed as dementia. More than 50 systems have been proposed for the evaluation of cognition, but there has been no consensus about a specific cut-off value for the diagnosis of mild cognitive impairment. An MMSE score of 24–27 was considered as mild cognitive impairment [29]. A prospective longitudinal study [52] conducted on Chinese patients found that among the 425 participants with 1529 person-years of follow-up, 14 patients with mild cognitive impairment progressed to dementia. The authors reported that metabolic syndrome, type 2 diabetes mellitus, central obesity, and hypertension were associated with a higher risk of progression to dementia. On the other hand, Koepsell TD et al. [53] reported that more than 15% of participants with mild cognitive impairment reverted to normal cognition after 1 year. Nevertheless, these patients remained at risk of future cognitive decline [53]. This issue deserves further investigation.

As for the sex-specific associations of metabolic syndrome with cognitive deficit, M. Schuur et al. reported that metabolic syndrome and insulin resistance were related to poorer executive function in women, but not in men [47]. In a longitudinal study conducted by Chanti-Ketterl, M., the higher the levels of metabolic dysfunction, the lower the levels of cognitive performance, and the above association was more predominant in women than in men [54]. Similar results were noted in a long-term longitudinal study [43], in which metabolic syndrome was associated with accelerated cognitive decline only in women. Our study demonstrated that women had a significant impact on the association between metabolic syndrome and cognitive decline (especially for the domains of orientation and memory), which was in accordance with the current understanding. This sex-specific correlation between metabolic syndrome and cognitive dysfunction could be explained by physiologic differences between men and women. First of all, patterns of cardiac remodeling and vascular aging differ between women and men [55,56]. With increasing age, women experience more concentric remodeling and diastolic dysfunction of the heart than men, and menopausal women have greater endothelial dysfunction and arterial stiffness compared with men. These sex differences in cardiac and vascular remodeling contribute to the increasing risk of cardiovascular disease in women, and in turn the risk of cognitive impairment. Moreover, ovarian dysfunction with estrogen deficiency in elderly women has been shown to be inseparably associated with metabolic syndrome [57] by resulting in atherogenic dyslipidemia [58], increasing intra-abdominal fat accumulation and insulin resistance [59]. In addition, a cross-sectional analysis demonstrated that women tended to receive inadequate treatment and had poorer control of several modifiable risk factors of cardiovascular disease compared with men [60]. Considering the physiologic differences and acquired socioeconomic disparity, women with metabolic syndrome may be more vulnerable to cognitive deficit than men.

This is the first study investigating the associations between the components of metabolic syndrome with various domains of MMSE in men and in women. Nevertheless, there were some limitations that need to be clarified. First, this is a cross-sectional study. The causation between metabolic syndrome and cognitive impairment cannot be confirmed. Some studies [61,62,63,64] proposed that prolonged poor-controlled metabolic syndrome was the key component leading to cognitive impairment. More longitudinal investigations that address the issue of gender differences in the association between metabolic syndrome and cognitive impairment were needed. Furthermore, all of the statistics in this study were collected from Taiwan Biobank. Considering that MMSE was not routinely conducted in all recruitment sites of Taiwan Biobanks and was available for less than 25% of the participants, only participants with information on MMSE were included for precise data analysis. Whether the study findings could be applied to other populations remains unclear. Moreover, dementia was defined according to the scores of MMSE < 24, regardless of the educational attainment, in this study. Given that people with a lower education level usually have poorer performance on the MMSE [49], the exact prevalence of dementia may be overestimated.

In conclusion, sex disparity has a significant influence on the association of metabolic syndrome with cognitive dysfunction. Women with metabolic syndrome are more likely to experience memory impairment or defective orientation. Considering that sex is a non-modifiable risk factor, women are encouraged to place a greater focus on preventing metabolic syndrome or managing other risk factors of cognitive dysfunction.

## Figures and Tables

**Table 1:** Characteristics of study participants according to sex.

Variables	Males	Females	*p*
Number of participants	7399	11546	
Age, years	64.2 (64.1, 64.2)	63.7 (63.7, 63.8)	<0.001
Body mass index, kg/m^2^	25.0 (24.9, 25.0)	24.0 (23.9, 24.1)	<0.001
Waist circumference, cm	88.1 (87.9, 88.3)	83.3 (83.1, 83.4)	<0.001
Systolic blood pressure, mm Hg	132.4 (132.0, 132.8)	127.8 (127.5, 128.1)	<0.001
Diastolic blood pressure, mm Hg	78.1 (77.9, 78.3)	72.6 (72.4, 72.7)	<0.001
Smoking, *n* (%)	1012 (13.7)	120 (1.0)	<0.001
Hypertension, *n* (%)	3688 (49.8)	4397 (38.1)	<0.001
Diabetes mellitus, *n* (%)	1612 (21.8)	1859 (16.1)	<0.001
Cardiovascular disease, *n* (%)	596 (8.1)	288 (2.5)	<0.001
Education level, *n* (%)			<0.001
≤6 years	735 (9.9)	2441 (21.1)	
>6 years	6664 (90.1)	9105 (78.9)	
Fasting plasma glucose, mg/dL	103.9 (103.3, 104.4)	99.3 (98.9, 99.7)	<0.001
HDL cholesterol, mg/dL	48.4 (48.2, 48.7)	57.9 (57.7, 58.2)	<0.001
Triglycerides, mg/dL	124.7 (122.8, 126.5)	116.8 (115.4, 118.2)	<0.001
MMSE	27.5 (27.5, 27.6)	27.2 (27.2, 27.3)	<0.001
MMSE < 24, *n* (%)	400 (5.4)	1042 (9.0)	<0.001
Metabolic syndrome, *n* (%)	2245 (30.3)	3514 (30.4)	0.905
Large waist circumference, *n* (%)	3033 (41.0)	7222 (62.5)	<0.001
High blood pressure, *n* (%)	4115 (55.6)	5155 (44.6)	<0.001
High fasting glucose, *n* (%)	3000 (40.5)	3417 (29.6)	<0.001
Low HDL cholesterol, *n* (%)	1645 (22.2)	3343 (29.0)	<0.001
High triglyceride, *n* (%)	1870 (25.3)	2495 (21.6)	<0.001

Data are presented as mean (95% CI) or *n* (%). HDL, high-density lipoprotein. MMSE, mini-mental state examination.

**Table 2:** Associations of metabolic syndrome with cognitive impairment (MMSE < 24) according to sex and education level.

	Univariate Analysis		Multivariate Analysis ^a^		
Independent Variable: Metabolic Syndrome	OR (95% CI)	*p*	OR (95% CI)	*p*	*p* for Interaction
Overall population	1.30 (1.15, 1.46)	<0.001	1.27 (1.13, 1.43)	<0.001	
Males	1.05 (0.84, 1.30)	0.685	1.02 (0.82, 1.27)	0.867	0.005
Females	1.54 (1.34, 1.78)	<0.001	1.48 (1.29, 1.71)	<0.001	
Education level ≤ 6 years	1.21 (1.03, 1.42)	0.018	1.21 (1.03, 1.41)	0.020	0.331
Education level > 6 years	1.08 (0.89, 1.30)	0.448	1.06 (0.87, 1.27)	0.579	

MMSE, mini-mental state examination. ^a^ Adjusted for age, smoking, and cardiovascular disease.

**Table 3:** Associations of individual components of metabolic syndrome with cognitive impairment (MMSE < 24) according to sex.

	Male		Female		
Independent Variables	OR (95% CI) ^a^	*p*	OR (95% CI) ^a^	*p*	*p* for Interaction
Large waist circumference	1.14 (0.92, 1.41)	0.226	1.25 (1.08, 1.46)	0.003	0.466
High blood pressure	1.01 (0.81, 1.25)	0.940	1.09 (0.95, 1.25)	0.235	0.562
High fasting glucose	0.89 (0.72, 1.11)	0.307	1.16 (1.00, 1.34)	0.046	0.054
Low HDL cholesterol	1.05 (0.82, 1.34)	0.711	1.16 (1.00, 1.34)	0.049	0.503
High triglyceride	0.90 (0.70, 1.16)	0.415	1.10 (0.94, 1.29)	0.251	0.187

HDL, high-density lipoprotein. MMSE, mini-mental state examination. ^a^ Adjusted for age, smoking, cardiovascular disease, and education level.

**Table 4:** Associations of metabolic syndrome with five domains of MMSE according to sex.

	Males		Females		
Independent Variables	OR (95% CI) ^a^	*p*	OR (95% CI) ^a^	*p*	*p* for Interaction
	Impaired orientation				
Metabolic syndrome	0.96 (0.79, 1.16)	0.661	1.21 (1.07, 1.37)	0.003	0.039
Large waist circumference	1.02 (0.85, 1.21)	0.868	1.19 (1.05, 1.36)	0.007	0.124
High blood pressure	0.92 (0.78, 1.10)	0.381	1.13 (1.00, 1.27)	0.044	0.066
High fasting glucose	1.00 (0.84, 1.19)	0.980	1.18 (1.04, 1.34)	0.009	0.122
Low HDL cholesterol	1.02 (0.83, 1.25)	0.880	1.14 (1.00, 1.29)	0.050	0.362
High triglyceride	0.93 (0.76, 1.14)	0.513	1.23 (1.08, 1.42)	0.003	0.025
	Impaired memory				
Metabolic syndrome	0.93 (0.82, 1.06)	0.289	1.12 (1.01, 1.25)	0.034	0.023
Large waist circumference	1.01 (0.90, 1.14)	0.845	1.07 (0.96, 1.19)	0.229	0.438
High blood pressure	1.06 (0.94, 1.19)	0.320	1.00 (0.90, 1.10)	0.943	0.467
High fasting glucose	0.88 (0.78, 0.99)	0.033	1.09 (0.98, 1.22)	0.113	0.006
Low HDL cholesterol	1.02 (0.89, 1.18)	0.740	1.13 (1.01, 1.26)	0.035	0.270
High triglyceride	0.93 (0.82, 1.07)	0.317	1.07 (0.95, 1.21)	0.285	0.138
	Impaired attention and calculation				
Metabolic syndrome	0.96 (0.83, 1.12)	0.597	1.09 (0.99, 1.21)	0.084	0.173
Large waist circumference	1.04 (0.90, 1.19)	0.599	1.04 (0.94, 1.15)	0.464	0.983
High blood pressure	0.92 (0.80, 1.05)	0.208	1.09 (0.99, 1.20)	0.068	0.038
High fasting glucose	1.06 (0.93, 1.22)	0.384	1.13 (1.02, 1.25)	0.017	0.502
Low HDL cholesterol	1.10 (0.93, 1.29)	0.257	1.04 (0.94, 1.15)	0.487	0.536
High triglyceride	1.03 (0.88, 1.21)	0.699	1.11 (0.99, 1.24)	0.081	0.503
	Impaired language				
Metabolic syndrome	1.08 (0.94, 1.25)	0.275	1.09 (0.97, 1.21)	0.142	0.767
Large waist circumference	1.29 (1.13, 1.48)	<0.001	1.14 (1.02, 1.27)	0.022	0.246
High blood pressure	1.00 (0.87, 1.15)	0.974	1.00 (0.90, 1.11)	0.949	0.938
High fasting glucose	1.03 (0.90, 1.18)	0.692	1.16 (1.04, 1.30)	0.008	0.109
Low HDL cholesterol	1.33 (1.14, 1.56)	<0.001	1.14 (1.02, 1.27)	0.023	0.142
High triglyceride	0.94 (0.80, 1.10)	0.414	0.98 (0.86, 1.11)	0.715	0.605
	Impaired design copying				
Metabolic syndrome	1.13 (0.90, 1.42)	0.283	1.41 (1.23, 1.62)	<0.001	0.093
Large waist circumference	1.05 (0.84, 1.30)	0.679	1.31 (1.13, 1.52)	<0.001	0.075
High blood pressure	1.02 (0.82, 1.27)	0.857	1.14 (1.00, 1.31)	0.051	0.344
High fasting glucose	1.25 (1.01, 1.55)	0.043	1.30 (1.13, 1.50)	<0.001	0.692
Low HDL cholesterol	1.09 (0.85, 1.39)	0.516	1.18 (1.03, 1.37)	0.021	0.515
High triglyceride	1.00 (0.78, 1.28)	0.996	1.19 (1.01, 1.39)	0.032	0.236

HDL, high-density lipoprotein. MMSE, mini-mental state examination. ^a^ Adjusted for age, smoking, cardiovascular disease, and education level.

## Data Availability

The data presented in this study are available upon request from the corresponding author. The data are not publicly available due to privacy and ethical restrictions.
